# Materia Medica and Therapeutics: With Ample Illustrations of Practice in All the Departments of Medical Science, and Very Copious Notices of Toxicology, Suited to the Wants of Medical Students and Practitioners

**Published:** 1851-07

**Authors:** 


### REVIEWS.

---

Art. IV.—*Materia Medica and Therapeutics: with ample illustrations of Practice in all the departments of Medical Science, and very copious notices of Toxicology, suited to the wants of Medical Students and Practitioners.* By Thomas D. Mitchell, A. M., M. D., Professor of the Theory and Practice of Medicine in the Philadelphia College of Medicine; formerly Professor of Chemistry and Pharmacy in the Medical College of Ohio; Professor of Chemistry and Materia Medica in the Medical Department of Transylvania University, Lexington, Ky.; Lecturer on Obstetrics and Diseases of Women and Children; Author of "Elements of Chemical Philosophy," "Hints to Students," &c., &c., &c. Philadelphia: Lippincott, Grambo & Co., successors to Grigg, Elliot & Co. 8vo pp. 738. 1850.

There have been quite a number of works written by American physicians on Materia Medica. No other department of medicine, we believe, has called forth so many treatises. If each of these had been an improvement upon its predecessor, the last must have reached far towards perfection—such perfection, at least, as is attainable in a progressive science. But, unfortunately, motion is not always progress; there may be much fluttering and only very slow and painful flight. In the history of the organic world, it appears that steps have sometimes been taken backwards—that instead of advancement in the series of creations there has sometimes been degradation. The youngest born have not been uniformly superior to the races created in an earlier age. And so, we fear, it turns out in our medical literature pertaining to Materia

Medica:—the last offspring of the press, to say the least of it, is certainly not the best.

The volume before us contains many things not noticed in the older works, as might be expected, for a very small man standing on the shoulders of a Goliath would see farther than the giant himself; but the additions, in every case, are not improvements. It has many passages that might be omitted without detriment to its fair proportions—a number of anecdotes, some sallies of humor, and occasional overflows of sentiment, very well, perhaps, before a medical class, for which it is evident enough they were originally prepared, but out of place, we cannot help thinking, in their present connection. But this gives the work variety, and, it may be, will add to its popularity. Whatever else it lacks, it assuredly cannot be said to want comprehensiveness. Besides the topics appropriate to such a treatise, physiology, diagnosis and hygiene come in for a share of the author's attention, the subject of poisons is discussed at considerable length, and the temperance reform receives a passing tribute. Almost every reader may find in it something to his purpose in, as the author expresses it, "*all* the departments of medical science;" and if this is not the way to make the most scientific book, it is the way to make a convenient and even a useful one.

The author has selected the alphabetical arrangement as the one which, after an experience of eleven years, he esteems as, upon the whole, the best. It unquestionably, with many objections, has its advantages, not the least of which is that all that it is necessary to say of an article is given under one head. Opium, for example, is disposed of in one place, instead of being distributed along under the various heads of narcotics, stimulants, diaphoretics, and antispasmodics; but on the other hand, when the reader wishes to learn what remedy will best fulfil the indications of the case before him, he feels the want of the common and more scientific arrangement of the materia medica.

Respecting the *modus operandi* of medicines, Dr. Mitchell inclines decidedly to the views of the vitalists. He does not deny that "medicines are absorbed and carried through the circulation, and thus exert their power," but he is averse to this explanation of their mode of action in most cases. Thus, when a rhubarb poultice applied to the abdomen of a child produces purging, it acts, he thinks, through "a nervous medium;" and he adds that "it is not necessary to look to cutaneous absorption for an explanation of the action of *any* remedy applied to the surface." The infinitude of nervous filaments, in his view, will account for all the effects observed in such cases. Now, it appears to us, this is making the nervous filaments account for a great deal. Tobacco is applied to the surface and it induces nausea and vomiting; a rhubarb poultice is spread over the abdomen and purgation is the effect; and again the surface is bathed in an atmosphere of sulphuretted hydrogen and the animal dies asphyxiated. If the nerves are answerable for this diversity of results, it must be owned that they have a complicated task to perform. There is no absorption through the skin, according to this theory, but all depends upon nervous influence; and so when the "bark jacket" breaks up an intermittent fever, or mercurial ointment salivates, or croton oil purges, it is done through the agency of the nerves! We might adopt the words of our author, in another place, and say, "if, perchance, any one can be found who cherishes such an opinion, most assuredly he is welcome to all the profit of such a notion."

Our author attaches some importance to the nose as a medium of medicinal impressions, and finds in this fact an argument against the use of snuff. "The man," he says, "who plasters his schneiderian membrane with impervious coats of this article loses one avenue, at least, by which remedies should find an entrance to the system." But it appears to us that the argument is not a good one. If the impervious coat of snuff keeps the medicine out of the system, is it not evident that it will keep out the poison of disease also, and does not the inveterate snuff-taker gain thereby quite as much as he loses? Upon the whole, we rather think our author has furnished the snuffer with a plea for the practice of "plastering over his schneiderian membrane," especially if his residence chances to be in a country subject to fever and ague. If the "organ of smelling" is a door through which malaria finds its way into the system, the more effectually the entrance is closed the better.

Dr. Mitchell "repudiates the doctrine of specifics" altogether, but nevertheless admits that there seems to be "an elective affinity of certain medicines for certain parts and organs of the body, and an aptitude in them for effecting specific changes." He does not hesitate to believe, for example, that ipecacuan induces nausea and opium drowsiness—that certain articles purge, and others create perspiration; but that quinine can be a specific for intermittent fever is beyond his power of believing. And yet he contends that typhoid is merely modified remittent fever and curable by quinine, and mentions "vinegar, saturated with common house salt" as "very beneficial in dysentery and scarlatina maligna." While "repudiating the doctrine of specifics" he refers to some specific on nearly every page of his book, and has one for half the ills that flesh is heir to.

On this point, Dr. M. has fallen into the error of those who would be "righteous over much." He has a becoming contempt for mere routine practice, but in warning his readers against this heresy, he has run into the opposite one, and is guilty of great absurdities. His sermon is continually at war with his text; his premises are contradicted by his conclusions; his details subvert and destroy his cardinal proposition. He sets out with denying the existence of specifics, and concludes by proving that almost every medicine acts somewhat in that character. And so it was obliged to turn out. All the testimony in the case is diametrically opposed to his dogma. If it is not ascertained that individual remedies exert a defined, *specific* influence, over morbid conditions of the human body—that, for instance, iron corrects anemia and quinine arrests an ague—there is nothing settled in medicine. We proceed, in fact, continually upon the theory of specifics. Every article of the materia medica is fitted to produce its peculiar influence. Each emetic, each purgative, every narcotic and diaphoretic—iodine, iron, mercury, all are, to a certain extent, specific in their action.

To one of our author's *specifics* we have decided objections—we refer to *carbonic acid* in consumption. He says:

"In phthisis pulmonalis, with great irritability of the lungs, diluted carbonic acid gas, taken by inhalation, often acted as a palliative, and it deserves further trial in this relation."

"Diluted carbonic" acid, but *how much* diluted? Dr. Mitchell is the author of a work on chemistry, and cannot have forgotten that this acid, even when very largely diluted with atmospheric air, still retains its poisonous attributes. Will he undertake to prescribe how much of it shall be mingled with the air to be respired by the consumptives? We ought to have precise rules with regard to the administration of so potent an article. The practice is on a par with that recommended by Beddoes, of sleeping in cow-houses to respire the air contaminated by the cattle, and is, in fact, the same revived. The old practice, we believe, was never pursued long or extensively, and it is not likely to be adopted in the shape now presented. We know not how it may strike other readers, but we confess it does seem to us that a writer is poorly employed vamping up and sending forth crude, exploded notions like this.

Our author directs the attention of his readers to the fact, that *sulphuric acid is blackened by cork.* At first, one is at a loss to understand why this is singled out from among the many properties of the acid, but the reason is soon made manifest—it affords him an opportunity to indulge in an anecdote. Mr. Randolph, in one of his rambling speeches in the Senate, once said, that "he would at any time go fifty yards out of his way to kick a sheep." Dr. Mitchell will go as far out of his way to perpetrate an anecdote. The following is the one told in connection with the fact that sulphuric acid is blackened by cork:

"A miserable creature called at my house many years ago, when I resided in a country village, introducing himself as a poor, unfortunate doctor, who had been engaged in a good practice in some part of Great Britain, but having been overtaken by misfortune, left for this country, in the hope of getting a livelihood. He begged me to make him a present of a lancet, as that would help him to a little money now and then for the operation of bleeding. I complied with the request of the poor creature, though he was by no means a deserving object. Some four or five days after, a letter came to my address from the same traveling doctor, in which he thanked me most cordially for the lancet, adding a prescription for cancer, with which I might "make a fortune." It was simply to blacken sulphuric acid with cork, and call the mixture "*Mitchell's Black Cancer Drop.*"

"Rather recently," he says, sulphuric acid has been recommended for the prevention and cure of colica pictonum. A good many years ago, it was announced as an antidote for lead poison, but M. Grisolle shortly afterwards stated, as the result of his experiments, that it possessed no powers of the sort, since which time but little has been said about it in that connection.

The subject of spirituous liquors, in its hygienic and moral bearing, is one of so much importance that we can tolerate a good deal of tedious commonplace in its discussion, but really we do not see why Dr. Mitchell should have gone gravely about proving to his readers that alcohol, as such, is an artificial product, and therefore "never a direct gift of Providence"—that it is an educt and not the product of distillation. We supposed that this truth had been iterated and re-iterated by temperance lecturers so often and so long, that, not only the merest tyros in medicine, but the dullest of the common people had come to understand it. But our author had got it into his old lectures and it must needs come out in his book. Nothing that he had there raked together can be kept back—not even the preposterous and silly assertion, that "a large majority of the sots have been made so by the doctors, through the agency of alcoholic medicines!" This, surely, is bad enough, but it is hardly equal to the following passage:

"Dr. Christison doubts the correctness of reported facts of diluted alcohol found in the ventricles of the brain, although asserted by several very respectable physicians in different parts of the world. But his reasons for being skeptical do not carry with them, to my mind, the slightest force. He might as well, at this time of day, set aside the conceded position that spontaneous combustion has been the fate of more than a hundred drunkards, and these scattered over the globe, by his notion, that the liquor must coagulate the blood, if so inflammable a fluid, as is alleged, pervades the whole body. But to me it would appear much more strange that some few, at least, of the great army of drunkards, should not have every living fibre so thoroughly saturated with that which for years has been their principal meat and drink, as to be susceptible of instantaneous combustion, in the manner so often heralded in the newspapers and scientific journals."

We make no comment on these extracts ; they require none. A writer, and he a physician, who asserts that "a large majority of the sots have been made so by the doctors," and holds that "every living fibre" of the human body may become so thoroughly saturated with alcohol as to be "susceptible of instantaneous combustion," cannot need to be refuted. He may be safely left to himself.

Dr. Mitchell is not quite so decided a believer in spontaneous generation as he is in spontaneous combustion, nevertheless he is a moderate advocate of that doctrine. He believes that, while intestinal worms are, for the most part, derived from an external source, they may be generated within the body ; and the fact, he thinks, is established by the old "practice of punishing criminals in Holland, by depriving them of salt entirely." When this cruel law was in force, it is said that worms were generated in incredible numbers in the bodies of its unfortunate victims ; but has it never occurred to our author that the fact admits of another explanation than the "formation" of the parasites by the unhealthy "mucous tissue?" Is it not possible, that salt either destroys the ova of the worms, or else prevents that condition of the alimentary tube upon which their development depends ? The farmer knows full well, that though he may deposit his seed, he is not sure of a harvest unless he has first prepared the soil for its reception. Each entozoon has its favorite habitat—some chosen animal body to which it attaches itself, and upon whose fluids alone it thrives and prospers ; and so it comes to pass that every species of the animal race has its peculiar parasites. The horse and his rider are not infested by the same intestinal worms ; fishes, birds, reptiles, the molluscous tribes, have each their own tormentors in the shape of epizootics.—And since a particular body is necessary to their existence, is it not likely that a particular condition of the mucous surface may be necessary to their development?

This explanation of the fact now fully established, that certain habits and modes of living are favorable to the production of entozoa, certainly appears to us not less satisfactory, and a good deal more rational than the one afforded by spontaneous generation.

We do not propose stopping to discuss this hypothesis, but one or two facts occur to us which bear so cogently upon it, that we believe our readers will pardon us for alluding briefly to them. Not only is each animal species, as we have said, infested with its own cimices and pediculi, but different nations of people have their peculiar parasitic worms. Thus, it has been established, that the Dutch, the Germans, and the English are subject to *Tænia solium*, and that the Swiss and Russians are troubled exclusively with *Bothriocephalus latus*, whilst the inhabitants of the French provinces adjoining Switzerland are infested with both species. The citizens of Dantzig suffer with tænia, those of Koingsberg, which borders upon Russia, with bothriocephalus; but as they sometimes travel beyond the borders of their respective countries, they may become the subjects of either. The case of the great anatomist, Soemmering, furnishes an example in point. He is known to have suffered with bothriocephalus, though a German; but it has been ascertained that he was in the habit of visiting occasionally a friend in Switzerland where that worm is indigenous.

What is the language that these facts speak? That "the mucous tissue" of a Dutchman's stomach generates one species of worms, and that of a Russian's another? This is the explanation of Dr. Mitchell and those who hold with him to the doctrine of spontaneous generation. We need hardly contrast its merits with those of the other solution of the difficulty—that different species prevail in different localities and there deposit their eggs to be swallowed by the inhabitants of the country with their food.

Considerable space is given by Dr. M. to the subject of water, but not more than it deserves. With all enlightened practitioners of the present day, he ascribes to this article great remedial virtues. It is curious, not to say humiliating, to remark how professional opinion has fluctuated on this point, and how old errors have been revived—how the cooling, and then the heating, and then again the cooling system has prevailed—but it is to be hoped that the rational practice in reference to the use of cold water in disease is now fully and finally established. Dr. M. seems to think there is special danger in the imprudent use of iced water. The contrary appears to be the fact, for fewer accidents occur where laborers have access to iced water than when spring or well-water is their drink. Such, at least, has been the experience of planters in Virginia, who have tried both with their operatives during the harvesting season in summer, and we have no doubt that it will prove to be the general experience.

The article on *nitrate of silver* is an interesting one, but contains some decidedly curious things. It is stated, for example, that "beginning with doses of the eighth of a grain, it may be gradually carried to *almost any quantity;*" and on the next page a case is reported of an epileptic who was cured by the continued use of the remedy, "but finally died of diseased liver and dropsy," and whose "viscera were all found marked by a blue tinge," particles of metallic silver being detected in the pancreas by Mr. Brande. Upon these particulars our author remarks:

"This fact proves quite conclusively the agency of the vital forces to decompose the most perfect salts, and that this power is far more potent than the merely chemical agencies of the animal economy."

Now, in the first place, we very much doubt the propriety of the remark, that the dose of nitrate of silver "may be carried to almost any quantity." Is there no danger to be apprehended from it? Dr. M. thinks not. He says he "should have no fears of poisoning a patient by thirty grains." We hope his readers would have many. No doubt the activity of the salt has been over-rated, and that, owing to the change which it undergoes in the stomach, it becomes comparatively inert; but are we to presume too confidently upon this change? Suppose it should not be converted into a *chloride*, but remain a soluble, caustic *nitrate*, what would be the result? If a few grains only should escape the change, will Dr. M. be answerable for the consequences?

But, admitting that the nitrate of silver becomes an insoluble, inactive chloride, in the stomach, or is reduced to its metallic state in the organs, and thus rendered harmless, what proof is there that the power by which the change is effected is "far more potent than the merely chemical agencies of the animal economy?" What are "the chemical agencies of the animal economy?" In what do they differ from the vital agencies? How has our author ascertained that they are of a subordinate character? We know very well that nitrate of silver is decomposed by a great variety of agents, and always, out of the animal economy, by an action purely chemical. Can our author, who is a chemist, conceive of its conversion into a chloride, or of its reduction to the metallic state, by any other agency? Is not the change, wherever effected, a chemical change? The very facility with which the decomposition is brought about, renders any other mode of explanation unnecessary, not to say absurd. Our author's philosophy on this point, we will do him the justice to say, is not as dangerous as his practice, but it is quite as irrational: neither strikes us as of a quality likely to gain much favor with his readers.

Under the head of *Arsenic*, Dr. M. makes the following singular remarks:

"It was at one time believed that arsenic spread on the earth's surface exerted a fertilizing influence, although not taken up by the vegetation grown thereon. It would seem, however, from the following statement, that the poison of arsenic is transmissible in vegetable matters, so as to exert a deleterious influence."

He then goes on to quote the account given in several English journals, of partridges poisoned by eating seed-wheat which had been steeped in a solution of arsenic.

Will Dr. M. do us the favor to inform us when and where the belief prevailed, that "arsenic spread on the earth's surface exerted a fertilizing influence?" We do not say that so absurd a belief never had existence, but we are sure that in the instance referred to by Dr M., the arsenic was not used for any such purpose. It was employed by the farmers in the form of a strong solution, with the view of preventing the ravages of the wire-worm on the wheat; and the practice is reported to have been eminently successful. Not only is the worm kept away by the poison, but the plant when grown up is also preserved against the smut. The practice has become very general, and the consequence is that in the districts where seed-wheat is thus treated, the poor partridges and pheasants suffer. And all this strikes our author as something very strange. "It would seem," he says, "that *the poison* of arsenic is *transmissible in vegetable matters!*" Certainly, it does so seem; not only the "*poison* of arsenic," but the very arsenic itself is "transmissible." Beyond all controversy, the wheat steeped in the arsenical solution imbibes the poison, and kills the unlucky birds that eat it. "This fact," our author thinks, "commends itself to the attentive study of medical men everywhere!" We should rather say, that it commends itself to the attention of those who are interested in the preservation of birds, especially the *game*-birds. We hope the cruel practice will soon come to an end in the old country, and never be introduced into ours. We are the friends of the feathered race, and are, moreover, believers, to some extent, in a principle of compensation. What the farmers gain on the one hand, we are persuaded they will lose on the other, to say nothing of the hazard to human health and life.

Our author is fully persuaded of the efficacy of the peroxide of iron as an antidote to arsenic. We are glad to find his faith holding out strong against the very powerful arguments by which the claims of iron to any such virtue have been opposed on chemical grounds. If we can only succeed in showing that the peroxide does save the subjects of arsenical poisoning harmless, we can afford to let the chemical objections go. No matter whether, upon chemical principles, it *ought* to be an antidote or not; *is it?* That is the all-important question. We hope it will turn out to be so; but we are compelled to say that the proof of it is not so convincing to our minds as it is to Dr. Mitchell's. Many cases, undoubtedly, might be cited in which persons took large doses of arsenic, and, following it by the antidote, escaped; but, unfortunately for the argument, many persons have taken the poison without the antidote and also escaped. Dr. Perrine, whose case Dr. M. has referred to, was saved from the enormous dose which he swallowed by mistake without the antidote. Dr. Meriwether gives the particulars of a case in the second volume of the *Transylvania Journal of Medicine*, in which recovery followed after ninety grains of arsenic had been swallowed by a young man, when as yet the oxide of iron had not been spoken of as an antidote. It would be easy to multiply instances of this sort, and in reviewing them in connection with the question of the antidotal powers of iron in arsenical poisoning, one cannot help certain misgivings that, possibly, the many favorable cases reported, may be so many examples of the error of ascribing to mere coincidence the relations of cause and effect—the old mistake of assuming *post hoc* to be, *ergo, propter hoc.* Still we are far from meaning to discourage the trial of the antidote. The most that can be urged against it is, that it is simply useless; no one has ever pretended that it can do harm, and as long as so many cases in which it has seemed to prevent the virulent action of the poison are unconfronted by cases of an opposite character, we should hold on to it.

But Dr. Mitchell exhumes another antidote which, from the testimony adduced, as far as it goes, would seem to have as strong claims to our confidence as the oxide of iron. This is *tobacco*, which, in the opinion of our author, "when it comes in contact with the arsenious acid, forms with it a *tertium quid*," and "a sort of *compound poisoning*" is the result, "in which the original poisonous quality of both articles is lost." We refer to this opinion, not because we attribute to it particular value, but as being somewhat characteristic of the author. The efficacy of the remedy in the first place, rests upon very insufficient evidence; but Dr. M. assumes that it is a true antidote and thereupon launches out into bold speculations as to its modus operandi. He has no facts upon which to base his opinions, but an explanation, *per fas aut nefas*, must be given, and so he concludes that the two poisons come together and form a *tertium quid*—a sort of harmless "compound poisoning!" And upon the strength of this theory he comes to the momentous conclusion, that "an inveterate chewer of tobacco could not be saved by the exhibition of any quantity of the article!" "Here's fine philosophy, an we had the trick to see it." In these days of cautious deduction how refreshing it is to encounter such a spirit of bold philosophising. No reference to facts; no waiting for the slow processes of observation and experience, but a jumping to conclusions. First, "tobacco is an antidote to arsenic"—assumed upon the slenderest possible ground. "It acts by forming with the poison a *tertium quid*;" (no proof in the world of it.) Therefore, "an inveterate tobacco-chewer could not be saved by any quantity of the article.!" After writing this, we wonder that it did not occur to our dashing author to add, *quod erat demonstrandum!*

We quit the subject of arsenic with one single quotation more, from which it will be seen that our author has fully as much faith in the chemical tests as in the chemical antidotes for this poison. He says—

"The *antiseptic quality* and the *indestructibility* of the poison, render its discovery in the body almost a matter of certainty, *even though twenty years* should pass away before suspicion should lead to examination."

Does he really believe that the antiseptic qualities of arsenic will preserve the human body undecomposed for twenty years? But suppose the victim has died of the nervous shock, after the ejection of most or all of the poison, how then about the "certainty" of its "discovery?"

The author takes in *auscultation* in his treatise, "though not," as he expresses it, "in the common acceptation, an article of the materia medica." Is it, in any acceptation, common or uncommon, "an article of the materia medica?" But it pertains to medicine, and as Dr. M. evidently designed his work to be a *pandect* on the subject, this was a sufficient reason for introducing it. In the same spirit he devotes twelve long pages to the subject of *bathing*, which he discusses in all its relations, physiological, hygienic, and therapeutical; or we should rather say, that he fills those pages with a quotation from the treatise of Dr. Robertson on Diet and Regimen. The passage quoted is an excellent one and will probably be read with as much interest as any other in the book.

Dr. M. speaks of Belladonna as a preventive of Scarlet

Fever in terms of decided approbation. He says, "I have employed the extract with such decidedly *preventive* efficacy that it would be folly or madness to doubt." Besides warding off the alarming disease, he adds, that he has "allayed the perturbation of nervous mothers effectually" by the prophylactic, and this alone he considers worth the trouble of the prescription. The prescription needs no extraneous recommendation if it can only be shown to be effectual. Establish the fact that scarlitina can be averted by the use of belladonna, and the practice will soon become universal. Our author directs three or four grains of the belladonna to be dissolved in an ounce of cinnamon water, of which a teaspoonful, more or less according to the age of the subject, is to be given to each child three times a day, for a week, when the medicine is to be intermitted for two or three days and then resumed as before.

Dr. M. is staggered by the practice of M. Mouneret, who administers the *nitrate of bismuth* to his dyspeptic patients in tablespoonful doses. He is not afraid of "*any quantity*" of the *nitrate of silver*, but when it comes to *bismuth*, he exclaims, "really it does seem to us, that such wholesale administration looks very like an argument in favor of the poetry of homœopathy." Of the two, we confess, we should declare for the bismuth practice.

*Bloodletting* is noticed at considerable length and a long letter is copied from the *New York Journal of Commerce*, in which Dr. Evan, of Sevoor, in the Deckan, as long ago as the year 1818, recommended that remedy in Asiatic Cholera. Could not our author, in all his reading, have found some testimony on that point a little more recent, and nearer home? Why go back to the annals of that early period, and of that remote region, when we have since had cholera in our midst and under our own eye?

Hitherto we have given our readers but few extracts from this work. The following is characteristic and, we believe, will prove amusing. Our author is treating of *bread pills*, or, as he learnedly styles them, *pilulæ panis*, concerning which he remarks—

"The philosophy of the use of bread pills as a remedy is more complex than some may imagine The prescription is an exceedingly simple thing in itself considered, and owes its success not so much to any inherent medical property as to the effect induced on the mind of the patient by the contingencies of the case. In other words, we accomplish good results with bread pills, by inspiring the *patient* with *confidence* in the means, and in our wisdom of the device. His confidence is essential to salutary results. Without it, we prescribe in vain.

The following case, which occurred during my pupilage, may serve as an illustration. A female consulted my preceptor (a worthy member of the Society of Friends), in relation to a disease of long standing. It was plainly a hypochandriacal affection, protracted and made worse by her sedentary habits. There was no evidence of organic disease in the system, and the good lady pretty clearly stated the case, by announcing her desire to have something prescribed that would *settle her nerves*.

The doctor listned to a long story with a pretty large measure of the patience of Job, but very soon decided what was to be done. He whispered in my ear to get some soft bread and make a box of forty pills, well scented with the oil of anise, and the suggestion was promptly carried out. With the box in his hand, the physician thus addressed the patient :—"My friend, I think my student has made thee some pills that will be of great benefit, if taken according to my directions. Hast thou a clock or a watch at home?" The lady gave a negative reply. "Thou canst borrow a watch of a neighbor, perhaps?" "Yes, I think I can," was the rejoinder. "Well then," said the doctor, "precisely at nine, twelve, and three o'clock in the day, three of these pills must be taken. Mind and be particular to the minute, or I cannot give thee any encouragement. Everything now will depend on thy self. Do as I direct, and call when the box is empty and let us know the result." The patient's whole mind was concentrated on a single point, her confidence in the doctor was full and complete. She retired under the deep conviction that the doctor had fully analyzed her case, and that the pills would cure her.

After the lapse of some days the patient was again at the office, and could scarcely find words to express her gratitude to the doctor. She declared that she had been improving every day since she began to use the pills, and now felt better than for ten years past. The box was renewed once or twice, and the recovery was complete."

On the subject of blistering some judicious remarks occur. Dr. M. insists upon their application at the proper time, which is all very well, and, we suppose, is very well understood, but his phraseology, in one place, is rather peculiar. Speaking of applying a blister to the chest of a man in pleurisy, with a full bounding pulse, he says, "fortunately, it would not act at all under such circumstances" or if it did, "its tendency would be to make the matter worse."

Referring to *Chloroform*, he says, "the notoriety acquired by this article as an *anæsthetic* agent entitles it to a short notice here." Now, with all due deference, we submit whether, if it had acquired no notoriety as an anæsthetic agent, the success with which it has been employed in a variety of diseases would not have entitled it to a good deal more prominent place than has been given to it in his book? We venture to express the opinion that if the reference to *bread pills*, *cheese*, and a number of such like matters had been made much shorter, or omitted altogether, and the article on choroform had been made much fuller than it is, the volume, without any increase of bulk, would have been rendered more worthy of the present state of the science. The reader, from the brief notice of it given by Dr. M. would be left to the conclusion that the value of chloroform in surgery is still a doubtful question. It has "been resorted to by *some* distinguished surgeons" he says, but adds, that "the professional mind is by no means agreed in regard to the practice"; and ventures the prediction that "in ten years chloroform will be discarded as an obstetric medicine." It would have been as well, perhaps if he had spared himself the exercise of his prophetic gifts and indulged a little more freely in historical details. His readers are fully as much interested in knowing what has been done with the remedy, as in learning what he imagines is to be its destiny. If he would allow us to offer a friendly suggestion, it would be that vaticination is somewhat out of place in a sober work on materia medica, and that what medical men are especially interested in understanding is how far the remedial agents submitted to their notice have been found efficacious in practice. In a word, they ask for facts, not conjectures or predictions.

The profession cannot be otherwise than deeply obliged to our author for the discovery announced by him, that "typhus and typhoid fevers are essentially and in fact *remittents*," and as curable in their earlier stages as remittents or intermittents by sulphate of quinine—that the only reason why the medicine does not cure them in the advanced stage" is "the establishment of lesions in the bowels." We fear the common experience of practitioners has been just the opposite of this, and that they have found the typhoid a disease quite other than a remittent or intermittent fever. It is easy to dogmatise, but bold assertion is of little worth in the absence of all proof. If typhus and typhoid fevers are so curable in their incipient stages, it must be owned that physicians have been slow to make the important discovery; and the fact is all the more unaccountable for that the diseases have been so long known, and submitted to every conceivable mode of treatment. How happens it that they have not yielded, in their forming stage, to some of the plans employed? We confess, we distrust our author's testimony on this point. We fear his sanguine disposition has led him to assert what future experience will not bear out. But let the experiment be made—let quinine have a fair trial in the earliest stages of these refractory fevers, and if the sequel should establish the efficacy of the method the authors of it will receive the thanks of mankind.

Fourteen consecutive pages are devoted by our author to the subject of *climate*—a subject belonging much more properly to hygiene than to materia medica—the whole being copied from Copland's *Library of Practical Medicine.* We notice this fact, not because these extracts are not quite as good as any other part of his work, but because it is very much in this style that his book is made up. It seems to have been his purpose to make a *big book*, and to that end he has not only dragged in topics from every department of the healing art, but levied freely upon authors all around, many of whom certainly are not so inaccessible as to have afforded him a pretext for copying scores of their pages. Why, for example, except to swell the size of his volume, take ten pages from *Williams* on *cod-liver oil*, or six from the *London Lancet*, on the tonic action of nitric acid, or these fourteen pages from *Copland*, on climate, or a dozen from *Robertson*, on bathing? As to the miscellaneous character of his topics we have had some illustrations. Among those introduced in the remaining portion of his treatise are the following: "compound poisoning," the "uses of cotton," "depuration of the blood," "regulation of diet," "elimination," "philosophy of vomiting," "fish-poison," "the causes of milk-sickness," "tinctures not necessary," "epilepesy cured by trephining," &c., We venture the belief that such a jumbling of subjects has not been often seen except in a "Receipt-book." The author, perhaps, hoped by making his work a sort of *encyclopedia* of the medical sciences to win every class of readers, or possibly he may have been ambitious to make a show of universal learning. But by whatever motive influenced, our belief is that he has not acted judiciously in forming such a salmagundi, and will find in the end that he has missed his aim.

Before bringing this article to a close we must favor our readers with another extract which is eminently characteristic of the work. Dr. Mitchell is speaking of *mesmerism* which he dismisses in the following manner:

"It may be expected by some that a considerable space would be allotted to the vaunted miracles of *animal electricity*, or *animal magnetism* or *mesmerism*. But the vagaries of this subject are so perfectly in keeping with the stale tricks and illusions of *jugglery* that I cannot regard them as entitled to a serious notice. There may be, and probably is, some truth mingled with the huge mass of delusion and falsehood; and the same might, with a show of propriety, be affirmed of the devil, albeit his accredited title is the father of lies. The developments of the mesmeric philosophy have been marked by a periodicity of type, coming and going by fits and starts, as the interest of the operatives prompted; and they are destined to annihilation, as they come in contact with cultivated minds and vigorous common sense. We cannot close these remarks better than by giving the following quotation from an article by Dr. Wigan (author of *Duality of the Mind*,) in the *London Lancet* for 1845. 'The treadmill is the only cure for mesmerism, at least of those who make it a means of extracting money from their dupes; as for the poor creatures who pursue the 'science' as amateurs, they must be left to themselves. We can prevent gaming-houses, but we cannot prevent domestic gambling. At any rate, while such exhibitions are tolerated, it is a shameful injustice to punish old women for giving poor servant girls delightful dreams by prophesying handsome and wealthy husbands, and taking their half crowns in exchange for the cheap bliss.'"

Now, our readers will bear us ready testimony, we have never shown any leaning towards mesmerism, but rather, perhaps, a temper inclined to hostility. We have discredited its high pretensions and steadily ranked it in the category of humbugs; but, we must say, that we find our sympathies for it somewhat stirred by such an assault as this. As we read it, we confess our feelings were somewhat akin to those of the benevolent Scotch divine, naturally recalled to our recollection by the language of the extract, who, after exhausting every other topic of prayer, proposed to his congregation to "*pray for the puir Deil.*" Mesmerism, as it pervades society, is monstrous enough, in all conscience, but give it its due—it is not as flagitious as Dr Mitchell would make it out. There is charlatanry enough about it, but it is not all a trick. Many of its alleged phenomena are too preposterous to be admitted, but many others must be received as unquestionable physiological facts. No one, we suppose, doubts the reality of the mesmeric sleep, or the blunted sensibility of persons when in that condition. Some other effects are as indubitable as these, and have their analogy in morbid states of the system well known to the physician. But even if the thing were worse than it is, (and it could hardly be worse since it has been married to "Spiritual Rappings,") such terms as are contained in this quotation are not those in which it becomes scientific men to assail it. With all its enormities, the respectability of its advocates gives it some claims to respectful consideration. We must do it justice; reject all that is false in its pretensions—brush away the bushels of chaff about it, and garner up the few grains of wheat that it has produced.

Touching *homœopathy* our author has some spirited remarks which are in better taste than many other parts of his book. They are as follows:

"Everybody who has read of the dilution of medicines, understands well enough what is meant by infinitesimal doses and infinitesimal practice. Rub a grain of opium with a hundred grains of white sugar, and the longer you rub, the stronger, medicinally, is the product. Take a grain of the mixture, add a hundred grains of sugar, and rub again. Of the mixture so made, take a grain and rub with it another hundred grains of sugar, and repeat the same process twenty times, and then determine, if you can, how much opium is in the final product of the mixture and trituration ; and some faint idea may be formed of the actual inherent power of *infinitesimal* doses, and of *infinitesimal* practice. We cannot waste more time on the subject. To make the effort would require a vast deal more patience than good old Job ever manifested or possessed. The man who can coolly swallow such a system, is ripe for the credence of any absurdity, no matter how impossible. In our view of the subject, it is really necessary for one to stultify himself, to become actually demented, or intentionally to play the knave ere he can deliberately attempt to reconcile such a system with common sense and the acknowledged laws of the animal economy. We may be mistaken, but cannot reach any other conclusion.

The attempt to justify these almost invisible doses, on the assumption that disease is *intangible, spiritual, ethereal*, and that the remedy must be kindred in its nature, is absolutely insane. Oh ! it would be well for many an aching heart, if the *croup* and *laryngitis* and *pneumonia* that have smitten their loved ones to the earth, could be made to appear as intangible, spiritual, etherial. Then might they find consolation in the fact that infinitesimal and intangible doses were heaven's only method of cure ; and that they failed because failure was inevitable. But the knife tells another story, when it discloses the frightful disorganization of parts most essential to life, aggravated and made positively incurable by the mad devotion to a moon-stricken philosophy."

With this extract we close our notice of Dr. Mitchell's volume, our estimate of which our readers will gather from what has been written. There might be a worse treatise produced on the subject; in many respects, we think, it is susceptible of great improvement. If re-cast and re-written, with many additions and a good many omissions, it is a work which might be recommended. Its greatest defect is a want of judgment, and next to that is the want of taste which is displayed on nearly every page. The latter is comparatively a venial offence, although style, even on scientific subjects, is a thing not to be wholly disregarded; but the other deficiency is a capital one in a work on therapeutics. In all candor we are compelled to say, that its doctrines, on many important points, strike us as radically unsound, and its practice as unsafe. Upon the whole, however, it is superior to the author's "*Chemical Philosophy*" published some twenty years ago, and we take pleasure in bearing so much testimony to its merits. It is something for a writer to have excelled himself.

The mechanical execution of the book is unexceptionable. It is correctly printed on good paper, and presents an agreeable appearance. Messrs. Lippincott, Grambo, & Co., are proving themselves worthy successors to our old friends, Messrs Grigg and Elliott. We hope their next publication will be of a character to enable us to speak more favorably of it, than with a good conscience, we could of this. Y.

